# Cryo-electron microscopy: an introduction to the technique, and considerations when working to establish a national facility

**DOI:** 10.1007/s12551-019-00571-w

**Published:** 2019-07-29

**Authors:** David Bhella

**Affiliations:** The Scottish Centre for Macromolecular Imaging, MRC - University of Glasgow Centre for Virus Research, Garscube Campus, 464 Bearsden Road, Glasgow, G61 1QH UK

## Introduction

Cryogenic-electron microscopy (cryo-EM) has recently emerged as a powerful technique in structural biology that is capable of delivering high-resolution density maps of macromolecular structures. Resolutions approaching 1.5 Å are now possible and maps in the 1–4-Å range inform the construction of atomistic models with a high degree of confidence. This new capacity for investigators to determine macromolecular structures at high resolution and without the need for crystallogenesis has led to an explosion of interest in adopting cryo-EM. Consequently, there is a large unmet need for both training in cryo-EM methods and the establishment of new cryo-EM facilities to support structural biology research across the globe.

The Scottish Centre for Macromolecular Imaging (SCMI) meets these needs by providing access to world-class facilities for cryo-EM research as well as an on-going programme to provide training in sample preparation, imaging and computational image processing for the Scottish structural biology communities. I was very excited then, when I received an invitation to participate in the *Biophysics and Structural Biology at Synchrotrons* workshop. To support this event, I gave lectures on specimen preparation for cryo-EM and my own experiences of establishing a national cryo-EM centre as well as a research talk describing our recent work on asymmetric reconstruction of icosahedral viruses (McElwee et al. 2018; Conley et al. 2019; Conley and Bhella 2019). We also delivered a short workshop on computational image reconstruction using Relion (Scheres 2012), and visualisation of cryo-EM maps using UCSF Chimera (Pettersen et al. 2004).

### Freezing protein preparations for Cryo-EM

In common with most structural biology methods, the preparation of purified macromolecular assemblies for cryo-EM is by far the most time-consuming and difficult stage of a project. The most common question asked by researchers entering the field, is “how much protein do I need?” The good news is that the answer to this question is considerably less than for most other biophysical methods. Nonetheless, cryo-EM does require more material than that which would be considered ‘a lot’ by most molecular biologists. Typically, we would advise investigators to aim for a preparation at concentrations greater than 1 mg/ml of purified protein, in a volume of at least 50 μl. By contrast, a crystallogenesis trial might be expected to require 500 μl of protein suspension at 5–10 mg/ml. For cryo-EM, it is also advisable to prepare material in a low-salt buffer, with minimal additives, to ensure good freezing and image contrast.

Protein suspensions are frozen on 3-mm-diameter transmission-electron microscope (TEM) support grids made from a conductive material (e.g. Cu or Au) that are coated with a carbon film with a regular array of perforations 1–2 μm in diameter. A total of 3–5 μl of sample is loaded onto the grid which is then immediately blotted with filter paper with the aim of creating a film of buffer/protein on the grid that, when frozen, will be thin enough for the electron beam to penetrate. Optimising the ice thickness is a vital step in sample preparation as thicker layers of ice increase the probability that the incident electron will undergo multiple scattering events and thereby reduce the image quality. In the case of extreme ice thickness, the electron beam does not penetrate at all. After blotting, the grid is rapidly plunged into a bath of liquid ethane—a very effective cryogen that freezes water with sufficient rapidity as to prevent formation of ice crystals. The formation of a vitreous layer of ice is the fundamental step in cryo-EM and preserves the target in a near-native state. The resulting vitreous ice layer with suspended protein molecules must then remain close to liquid nitrogen temperature (− 196 °C) during storage and imaging in the TEM to prevent phase changes to other types of ice that are not amenable to high-quality imaging and preservation of protein structure.

Working with frozen-hydrated specimens brings a number of challenges both for manipulations and imaging. When handling cryo-EM grids to load them into the microscope, exposure to atmospheric water vapour rapidly leads to frost buildup on the grid. Under the TEM, these ice crystals on the grid surface appear as huge boulders that completely block the electron beam. Thus, grids are kept under liquid nitrogen as much as possible to minimise frost contamination.

### Imaging frozen-hydrated preparations by cryo-EM

**Fig. 1:**
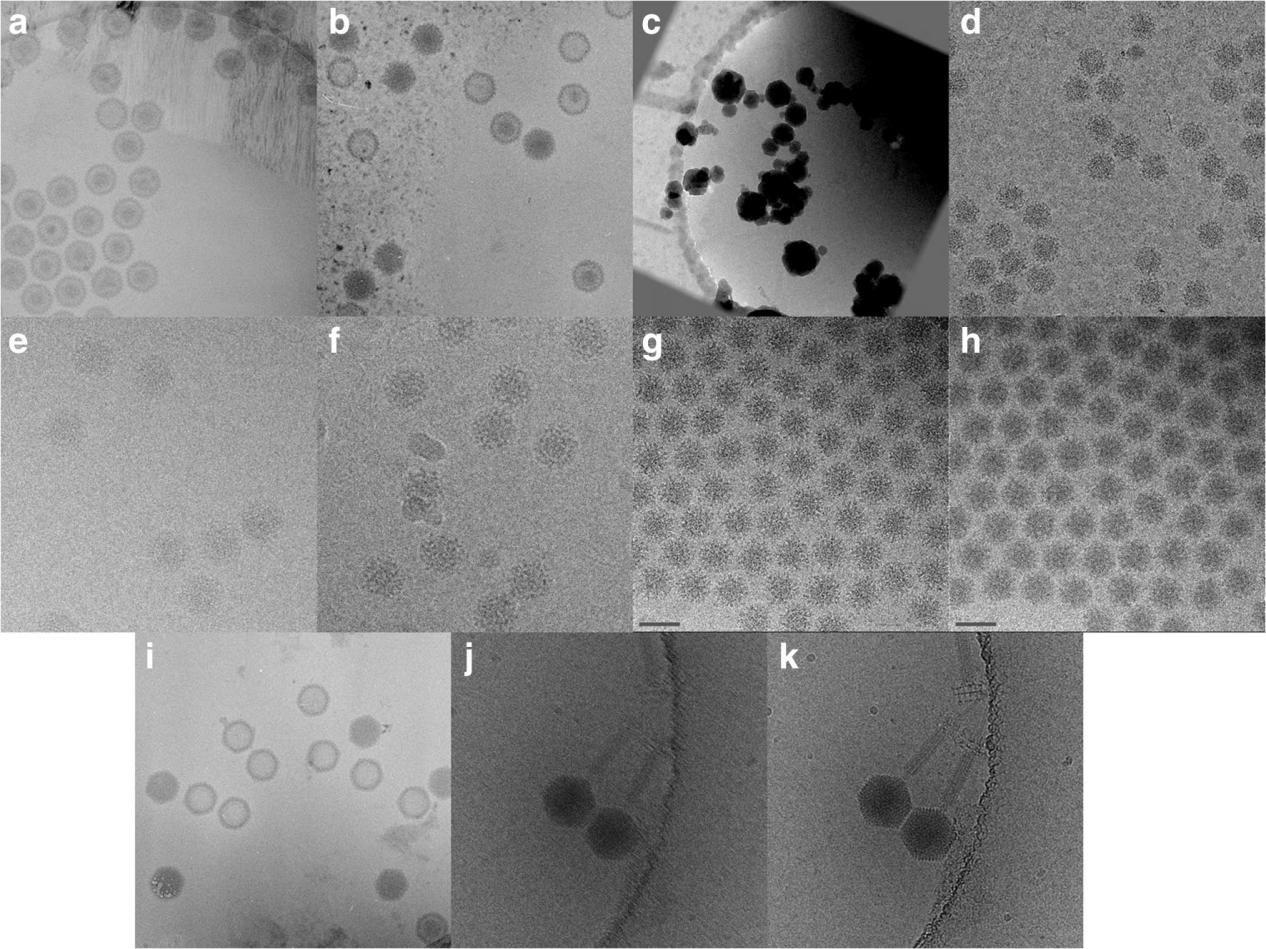
Gallery of images to show some common artefacts in cryo-EM. Insufficient rapid freezing leads to formation of hexagonal ice crystals (**a**), while warming of vitreous ice results in cubic ice crystals (**b**). Exposure to atmospheric water vapour during handling of frozen grids gives rise to condensation of large ice crystals (frost) (**c**), while contamination in the column or during transfer gives a more subtle-mottled contamination (**d**). To generate phase contrast, the TEM is defocussed. Close to focus images have lower contrast and finer features (**e**, 1-μm underfocus), while further from focus have greater contrast of coarser features (**f**, 3-μm underfocus). Cryo-EM samples are extremely sensitive to the electron beam. Radiation damage leads to destruction of high-resolution features (**g**, 10 e/Å^2^; **h**, 90 e/Å^2^), eventually bubbles of gas form in the ice (**i**). Specimen movement leads to blurring of information. Whereas previously images such as (**j**) would have been unsuitable for image reconstruction, motion correction of image ‘movies’ from modern direct detection devices rescues useful information (**k**)

When investigators first start to learn cryo-EM, they often struggle to distinguish good from bad images. Fortunately, in the course of a 25-year career in cryo-EM, I have had ample opportunity to accumulate a library of terrible images—for teaching purposes of course. Problems with ice conditions are common—insufficient rapid freezing leads to formation of hexagonal ice, while devitrification occurs when samples warm up, leading to formation of cubic ice (Fig. 1a, b). Various degrees of contamination may occur, and frosting at atmospheric pressure causes the above-mentioned ice crystal deposition, while contamination within the column or under low-vacuum conditions gives rise to a more subtle artefact—leopard skin ice (Fig. 1c, d).

Image formation in cryo-EM is primarily by phase contrast, although between 7 and 10% of image contrast is from amplitude contrast. Amplitude contrast, where an electron is scattered to such an extent it is removed by an aperture or energy filter along the path of the TEM column, is generally not considered, as the information obtained is usually low resolution compared with phase contrast. Phase shifts in elastically scattered electrons, relative to the undeflected electron wave, occur as a consequence of potential encountered while passing through the specimen and spherical aberration in the objective lenses. The TEM modifies the image of an object by a point-spread function (referred to commonly as the contrast-transfer function, which is the point-spread function in Fourier space). For in-focus images, the CTF attenuates low-resolution features preventing the identification of proteins or molecules due to the very low contrast between the target and the background. By increasing the defocus, phase contrast is generated across certain spectral ranges (note the improved contrast between the two images taken at increasing defocus, Fig. 1e, f). Modulation of information by the CTF under defocussed conditions however has the effect of eliminating, attenuating, or reversing contrast at higher spatial frequencies as well as delocalising image information; each point in the ideal image is convoluted with the oscillating point-spread function. Therefore, while higher defocus improves contrast, it comes at a cost of (i) increased fluctuations of the CTF, making accurate estimation and correction more difficult and thus limiting the resolution; and (ii) increased delocalisation of the image, requiring particle images to be extracted in larger windows to capture this information. During computational image reconstruction, it is possible to compensate for the effects of the CTF provided that some parameters of the TEM are known and that the defocus can be accurately estimated. Calculation of high-resolution 3D reconstructions depends on the collection of a dataset that covers a range of defocus values (typically between − 800 nm and − 2 μm underfocus) to compensate for the loss of information brought about by the CTF.

Frozen-hydrated preparations are extremely susceptible to radiation damage. A key element of cryo-EM is the use of low-electron dose conditions to minimise irradiation of samples prior to capture of images. Pre-calibrated imaging modes are established to (i) search for areas of interest at low magnification/dose, (ii) focus at high magnification/dose on an adjacent area and then (iii) record the image at high magnification and at a known dose. One of the several innovations that have elevated cryo-EM to a high-resolution method is the automation of this process. Correctly identifying radiation-damaged regions of the specimen is an important skill that new users also need to acquire (Fig. 1g–i).

Finally, new cryo-microscopists need to be able to identify imaging artefacts related to misalignment of the TEM and specimen movement. The latest generation of TEM cameras, direct detection devices, allows users to correct for these problems. The fast readout of CMOS-based cameras means that micrographs are recorded as ‘movies’—stacks of images that may be aligned and summed to correct for specimen movement (Fig. 1j, k). High-quality images also allow computational assessment of microscope alignment and automated correction of objective lens astigmatism and beam-tilt.

### Establishing a cryo-EM laboratory

Astonishing technological advances in cryo-EM have led to a revolution in structural biology. There is wide enthusiasm to access this method, principally from those already working in structural biology, and also from molecular biologists that may view structural biology as increasingly accessible. There is one inescapable reality however; cryo-EM infrastructure, while being cheaper than a synchrotron, is very expensive to buy and maintain. At a minimum, we might expect an initial outlay of ~ £6 M and recurring costs of ~ £500 k per annum to establish and support a cryo-EM facility comprising a single automated 300-keV cryo-TEM, and a single feeder microscope for project development. Thus, building cryo-EM capability requires a critical mass of investigators that are capable of raising the funds to ensure sustainability.

The SCMI was established as a consortium between four research-active universities: Glasgow, Edinburgh, Dundee and St. Andrews. These institutions provided both the collective need and capacity to support the necessary investment. A funding of £4 M for the primary research instrumentation was provided by the United Kingdom Medical Research Council. Additional funds and commitments totalling £1.8 M were provided by the partner institutions, Scottish Funding Council and a charitable trust (the MJM Smith trust).

To support the research needs of users, the SCMI has formed a network of feeder laboratories, each equipped with specimen preparation equipment and a 200-keV cryo-TEM and staffed by an experienced cryo-EM technician, to support project development.

A significant budget consideration in establishing a new cryo-EM facility is the suitability of the building in which the facility is to be housed. High-performance TEMs require a stable, vibration-free floor, high ceilings (typically 4–5 M), minimal electromagnetic fields and close temperature control. Consultation with engineers and architects at an early stage is therefore critical, and budget should be allocated for specialist air-handling and possibly electro-magnetic field cancellation. It is not uncommon for build costs to exceed £2 M. Ancillary equipment, including vitrification robots (~ £75 k), carbon coaters (~ £35 k), plasma glow-discharge devices (~£10 k) and specimen storage dewars should also be budgeted for. In the case of SCMI, the laboratory was established in the recently built Sir Michael Stoker building at the University of Glasgow, a state-of-the-art research facility that was designed to accommodate two high-end cryo-TEMs.

**Fig. 2:**
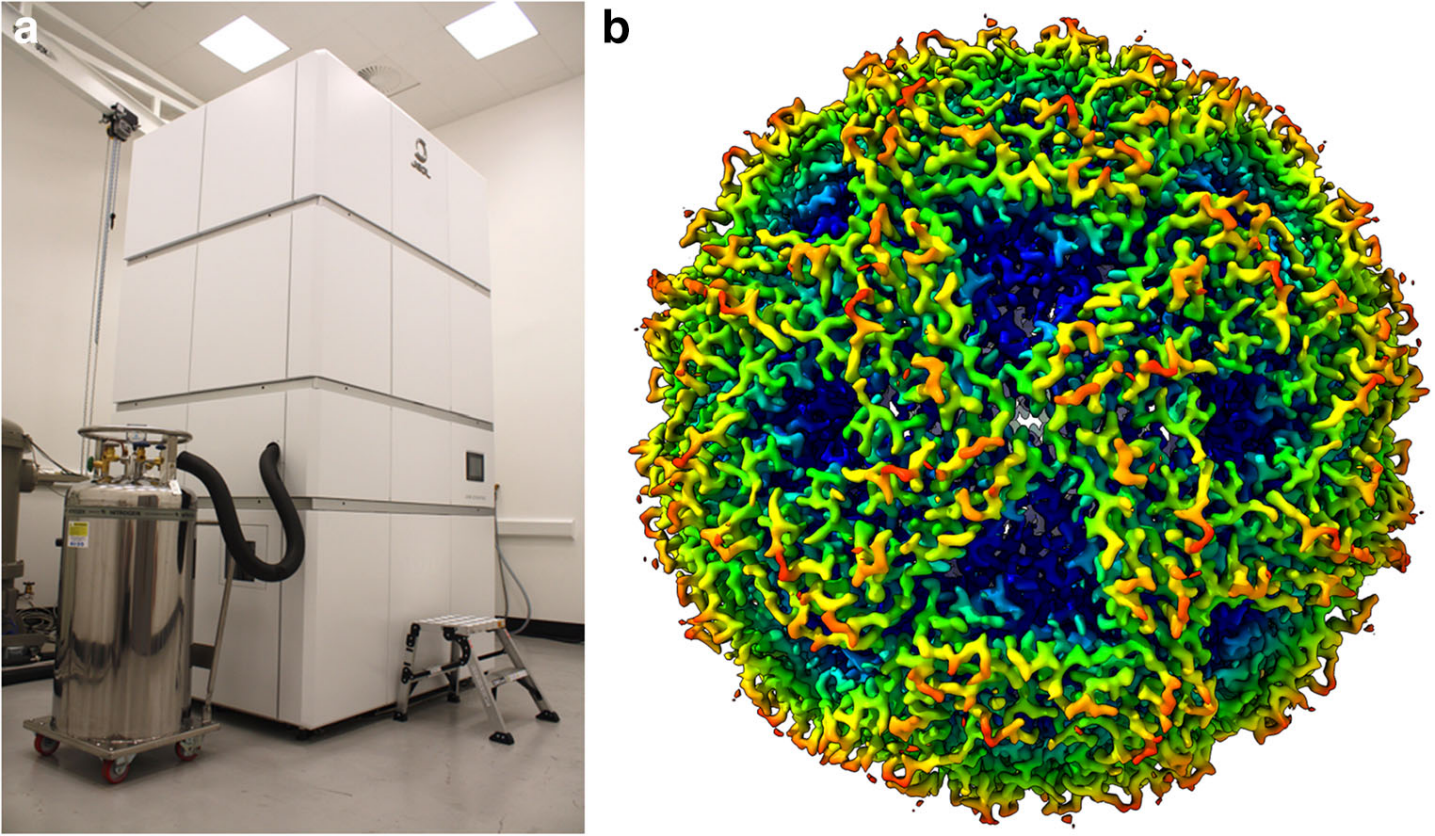
The Scottish Centre for Macromolecular Imaging JEOL CryoARM 300 (**a**). This newly designed instrument is currently undergoing commissioning; one of the first high-resolution structures solved on this machine is the 2.2-Å resolution structure of lumazine synthase

After a rigorous procurement exercise, we took a bold decision not to buy the market-leading automated 300-keV cryo-TEM: the Thermo Fisher Titan Krios. Instead we undertook to be among the first to buy a newly designed instrument: the JEOL CryoARM 300 (Fig. 2a). Several innovative technologies led us to conclude that this new machine had the potential to deliver superior performance. Firstly, the specimen autoloader—a robotic system for loading cryo-EM grids into the microscope—had several attractive design features. Up to 12 grids may be loaded, four at a time, held in cartridges by c-clips. ‘Clipping’ grids is very easy, limiting the risk of accidentally dropping grids in the column, due to incorrect mounting. Grids are held in a cold-storage area that is infrequently vented to atmosphere, reducing the risks of contamination. Grids may be stored for weeks or even months, if required. Obviously, the source of electrons is a vital component for any TEM, and the JEOL CryoARM comes equipped with a novelty in the biological sciences: a cold field-emission gun (CFEG). While CFEGs deliver the brightest, most coherent beam of electrons with a very narrow energy spread and the smallest beam source, compared with other more conventional electron guns (such as the Schottky FEG), they have historically suffered from lower beam stability caused by fluctuations in the pressure in the TEM column. I am pleased to report that the new JEOL CryoARM has overcome this issue (see below) and so has an extremely optimal electron source for high-resolution data acquisition.

We also chose to buy our camera from a company that is not market dominant. The Direct Electron DE-64 offers a very large CMOS-based direct-detection device that has a level of versatility not available from rival products. The large images are very well suited to both conventional single-particle cryo-EM where many thousands of views of macromolecular complexes are averaged together to produce the final reconstruction, and also for electron tomography, the 3D visualisation of a single unique object such as a cellular organelle or an enveloped virus particle. In such cases, the specimen is rotated in the microscope to produce a tilt series of images that can be processed to yield a three-dimensional density map. To obtain the very highest quality image data, direct detection devices may be operated in electron counting mode. To achieve this, the TEM is configured with a very weak beam, such that single-electron events on the camera may be recorded within one read-out cycle. This has the advantage that any ambiguity concerning the number of electrons that contributed to the accumulated charge in a single pixel is removed. When operated in electron counting mode, and with 2× pixel binning (reducing the read-out size), the DE64 delivers an excellent detective quantum efficiency (DQE, the ratio between the ‘true’ information in the object to how faithfully that information is captured by the camera).

Being early adopters of two newly designed instruments that do not yet have a large customer base has come with some inevitable challenges. Although some difficulties remain, one year into the installation process, we are preparing to begin to deliver a service.

First data from the microscope indicate that it is capable of delivering excellent quality, for example a reconstruction of the icosahedral enzyme complex lumazine synthase has been calculated at 2.2-Å resolution (Fig. 2b).

### Perspectives

Attending the *Biophysics and Structural Biology at Synchrotrons* workshop was a tremendous opportunity to meet with many inspiring and motivated early career researchers working in Africa on projects that address the needs of that continent. Meeting scientists from across the globe is one of the most rewarding aspects of a career in science, and it is always fascinating to learn about the research interests and priorities of different nations. Enthusiasm for biophysical methods and structural biology was evident among the young scientists attending the workshop, and I hope that the introduction to the range of methods available to them through European national facilities will lead to many fruitful collaborations.
